# A possible approach to overcome the systemic toxicity of azathioprine (AZA) through nanoemulgel

**DOI:** 10.17179/excli2025-8223

**Published:** 2025-03-19

**Authors:** Bikash Kumar Sah, Faiz Ahmad, Sachin Kumar Singh, Ankit Kumar

**Affiliations:** 1 School of Pharmaceutical Sciences, Lovely Professional University, Phagwara, Punjab, India

## ⁯⁯

The immunosuppressive medication azathioprine (AZA) is a prodrug, converts to metabolite 6-mercaptopurine which functions as a purine synthesis inhibitor. It is reported to control the proliferation of T and B lymphocytes which are considered as one of the causes of Rheumatoid Arthritis (RA). The use of AZA for the treatment of RA reduces the production of autoantibodies, tumor necrosis factor-α (TNF-α), and interleukin synthesis from immunosuppression. This leads to the reduced chronic inflammation. Additional research shows that patients experience significant symptom relief alongside the joint stabilization when taking AZA. However, oral non-targeted administration of AZA leads to the suppression of host's immune response which makes the patient susceptible to other infections and neoplasms (Rios-Usuga et al., 2024[5]).

The World Health Organization reported that around 17.6 million people suffered from RA in 2020 and the number is expected to rise to 31.7 million by the year 2050. Men, women and children can develop RA, yet this disease appears 2-3 times more frequently among women than other gender groups. The frequency of the condition grows with age while its main onset occurs during the period between 60 and 70 years of age (Black et al., 2023[1]).

A key challenge with AZA treatment stems from its low aqueous solubility and the resulting functional barriers to efficient drug absorption and, stable therapeutic concentrations in regular oral administration methods. Poor water solubility of AZA, delays treatment effectiveness and produces irregular drug amounts in the body which require increased dose amounts which tends to increase in the systemic toxic events such as hepatotoxicity and destruction of bone marrow (Morisset et al., 2017[3]). 

Oral AZA treatment for RA leads to gastrointestinal problems like nausea, vomiting, and diarrhea while reducing bone marrow function to cause leukopenia, anemia, thrombocytopenia, hepatotoxicity, hypersensitivity reactions, and increased infection susceptibility. Extended use of treatment increases the potential for users to develop both lymphomas and skin cancers. Patient monitoring of both blood counts and liver function plays a critical role in the care so patients must promptly notify healthcare providers about experiencing any strange symptoms. Clinicians must adjust doses or withdraw when serious adverse events arise particularly among patients who have underlying medical problems (Janakiraman et al., 2018[2]).

A site-specific delivery of AZA through nanoemulgel is expected to effectively resolve the difficulties associated with the therapy. The nanoscale droplet structure of the proposed formulation will improve AZA solubility followed by the greater absorption and improved therapeutic efficacy with reduced dose. Nanoemulgel with gel bases help deliver medications to inflamed joints with sustained release properties that lower bloodstream exposure resulting in reduced side effects and better clinical results (Movahedi et al., 2016[4]).

### Nanoemulgel of azathioprine

Drug delivery system based on nanoemulgel provides superior treatment of RA for AZA through improved drug permeability, localized distribution and better solubility. Ascendant drug delivery models feature nanoemulsion to enhance penetration while employing gels to maintain drug concentration at target inflammation points. Systemic absorption is minimized through this treatment method thus reducing medical adverse responses associated with oral or parenteral route administration. Direct application of AZA nanoemulgel enables effective joint-targeted inflammatory control while reducing the systemic drug levels which enhances patient adherence and provides long-term efficient management for RA. Nanoemulsion droplets remain very small which permits them to penetrate deeper tissues to achieve optimal therapeutic outcomes. Nanoemulgel provide patients with a comfortable non-invasive application that represents an advanced method for RA treatment while replacing conventional drug delivery systems.

Overall, the novel nanoemulgel delivery system improves drug localization in target areas while reducing the systemic drug toxicity along with their associated side effects. The formulation enhances drug solubility and bioavailability with improved penetration features that help patients effectively manage RA. The simple application method of this procedure drives better patient cooperation and provides safer performance than established treatment approaches. The technique represents a substantial improvement in RA treatment approaches.

## Conflict of interest

The authors declare no conflict of interest.
